# The cortical responses to evoked clinical pain in patients with hip osteoarthritis

**DOI:** 10.1371/journal.pone.0186400

**Published:** 2017-10-30

**Authors:** Mikkel Gram, Joachim Erlenwein, Frank Petzke, Deborah Falla, Michael Przemeck, Miriam I. Emons, Michael Reuster, Søren S. Olesen, Asbjørn M. Drewes

**Affiliations:** 1 Mech-Sense, Department of Gastroenterology and Hepatology, Aalborg University Hospital, Aalborg, Denmark; 2 Coaze IVS, Aalborg, Denmark; 3 Pain Clinic, Department of Anesthesiology, University Hospital, Georg-August-University of Göttingen; Göttingen, Germany; 4 Department of Anesthesiology and Intensive Care, Annastift, Hannover, Germany; 5 Clinical Institute, Aalborg University Hospital, Aalborg, Denmark; University of Akron, UNITED STATES

## Abstract

**Background:**

Experimental models have been used extensively to evaluate pain using e.g., visual analogue scales or electroencephalography (EEG). Stimulation using tonic pain has been shown to better mimic the unpleasantness of chronic pain, but has mainly been evoked by non-clinical stimuli. This study aims to, evaluate the EEG during clinical pain in patients scheduled for total hip replacement with control and resting conditions.

**Methods:**

The hip scheduled for replacement was moved by the examiner to evoke pain for 30 seconds while recording EEG. The control condition entailed movement of the opposite hip in a similar fashion and holding it for 30 seconds. In addition, EEG was recorded during the resting condition with open eyes. The relative spectral content was calculated from the EEG as well as functional connectivity using phase-lag index for frequency bands delta (1–4Hz), theta (4–8Hz), alpha (8–12Hz) and beta (12–32Hz). A mixed model was used for statistical comparison between the three recording conditions.

**Results:**

Spectral content differed between conditions in all bands. Functional connectivity differed in delta and theta frequency bands. Post-hoc analysis revealed differences between the painful and control condition in delta, theta and beta for spectral content. Pain during the hip rotation was correlated to the theta (r = -0.24 P = 0.03) and beta (r = 0.25 P = 0.02) content in the EEG.

**Conclusion:**

EEG differences during hip movements in the affected and unaffected hip appeared in the spectral beta and theta content. This was correlated to the reported pain perceived, pointing towards pain specific brain activity related to clinical pain.

## Introduction

Evaluation of pain in experimental and clinical studies relies mostly on subjective pain ratings such as the visual analogue scale [1,2]. However, due to the shortcomings with subjective methods a quantifiable and objective method, both for evoking pain and for evaluation—preferably linked to the physiological processing of pain—is needed [2]. Experimental pain models attempt to control the painful stimulus intensity, so as to standardize and quantify the state of the pain system [1]. A myriad of models exist, each with their own advantages and disadvantages depending on the application [1]. Although the input to the models (e.g., pain intensity, modality and temporal pattern) is highly standardised, the output (e.g., subjective pain assessment, electrophysiological and imaging methods) has more variability and needs improvement [1].

Electroencephalography (EEG) has been used for objective quantification of cortical processing of nociception in both healthy volunteers and patient populations [1,3,4]. EEG can be recorded either during rest or painful stimulations. The most common method to assess the pain response by EEG is evoked brain potentials, which measure the cortical responses to repetitive short, phasic painful stimuli. However, this method has drawbacks since the phasic stimuli poorly reflect clinical pain conditions [5], and findings also indicate that major parts of the EEG response are in fact not pain specific but possibly related to saliency [6,7]. In contrast, tonic pain stimuli may better mimic clinical pain conditions due to the persevering nature of the pain stimulus. However, tonic stimulation, has mainly been used in studies of healthy subjects and evoked by a non-clinical stimulus (e.g. the cold pressor test) [5]. Therefore, investigations of the continuous EEG response during evoked and controlled clinical pain conditions are of great interest.

Continuous EEG has been investigated in the clinic in various chronic pain conditions [3,8] and recent developments of EEG analysis have allowed advanced assessment of EEG networks through functional connectivity [9]. However, chronic pain imposes the limitation that no pain-free control condition can be recorded to compare to the painful condition, and the analysis is usually limited to comparison to healthy volunteers. So far, little work has been done using *evoked* clinical pain and no studies have compared the evoked pain responses to non-painful clinical stimuli [10].

We hypothesized that pain elicited by standardized and controlled hip rotation in patients with hip joint osteoarthritis would induce changes in the EEG which are associated with the sensation of pain. To investigate this the study aims were: a) to evaluate the EEG response in patients with hip joint osteoarthritis by evoking clinical pain from rotating the affected hip in a controlled manner (i.e. painful condition), b) to compare the EEG response during evoked pain to the EEG recorded during controlled rotation of the contra-lateral less or unaffected hip (i.e. control condition) and the EEG recorded during resting state (i.e. resting state condition).

## Methodology

The study was conducted by the Department of Pain Medicine, Clinic for Anesthesiology, University Hospital, Georg-August-University of Göttingen, Germany and the Department of Orthopedic Surgery at the Orthopedic University Hospital of the Medical School Hannover, Germany. The study was approved by the Ethical committees of the University Hospital of Göttingen (No 19/2/13) and the Medical School Hannover (No. 1483–2012). The study was conducted according to the recommendations of the Declaration of Helsinki. Patients provided informed written consent.

### Study subjects

Patients with osteoarthritis admitted to the Orthopaedic University Hospital Annastift in Hannover for scheduled total hip replacement were recruited between April and August 2013.

Inclusion criteria were patients above 18 years of age and able to give informed consent. Exclusion criteria were 1) severe neurological disease, which might interfere with the EEG recordings or experimental pain testing (including dementia); 2) Severe psychiatric disease such as major depression or schizophrenia or active drug abuse; 3) High dose of preoperative opioid therapy (> 30 mg/day oral morphine equivalent); 4) participation in other studies conducted at the same time.

### Study procedures

All clinical tests were conducted in the afternoon by the same examiner (MR). Patients were placed on an examination bed in a semi-recumbent position, and the EEG cap was mounted on their head with conducting gel inserted for each electrode. Resting EEG (see later) was recorded followed by EEG recording during the painful condition (affected hip) and lastly, the control condition (unaffected hip).

### Medication

The patients preoperative pain medication (type and dosage) was noted and the Medication Quantification Score, which is a reliable and validated score for quantifying analgesics, was calculated in order to obtain a comparable metric for different analgesic classes [11]. It was calculated for each non-opioid and opioid based on weights assigned by medication class and dosage level (level 1 = sub-therapeutic dosage and/or on demand, level 2 = <50% of the daily dosage, level 3 >50% of the daily dosage, level 4 = over dosage) using the 1998 detriment weights [12]. These scores were summed to provide a quantitative index for analgesic usage suitable for statistical analysis. In addition, percentage of patients on opioids and average dose were recorded.

### EEG recordings

EEG was recorded from a 34-channel cap (34ch prewired cylindrical Ag/AgCl electrodes, MEQNordic A/S, Jyllinge, Denmark) and amplified on a Nuamp system (NuAmp, Neuroscan, El Paso, TX, USA) and recorded for later analysis (Neuroscan 4.5, Neuroscan, El Paso, TX, USA). The cap was placed symmetrically in a standardized position 3 cm above the nasium. Electrode gel was applied into each of the recording channels to reduce the electrode impedance below 5 kΩ. Recordings were done in a dimmed light room with all unnecessary electrical equipment turned off to avoid 50 Hz contamination. Sampling frequency was 1000 Hz.

### Resting EEG

Patients were instructed to not speak or move as well as minimize eye-blinking during the recording. Open or closed eyes were alternated between in 4 sequences of 2.5 min, starting with eyes open. For this study, only the first recording of 2.5 min eyes open was used.

### Hip rotations

The pain detection threshold by rotation of the hip was determined by the patient while the examiner brought the hip scheduled for replacement into a 90°flexed position with knee flexion of 90° where it was held shortly. Then, internal rotation was performed until the pain threshold was reached. Next the EEG recording was started and the hip rotated slightly further and maintained in this painful position for a duration of 30 seconds. Afterwards the patients were asked to rate their pain on a numerical rating scale (NRS) from 0–10. The procedure was repeated for the less or non-affected hip serving as a control condition; EEG was recorded for 30 seconds and pain was rated using the NRS. In patients who could not tolerate this choreography (i.e. with severe clinical pain already with hip flexion), hip flexion was used as the clinical stimulus with the same procedure for EEG recording. In a few subjects external rotation served as pain evoking stimulus. In all these cases the less or unaffected was stimulated in the same way as the affected side.

### Pre-processing

The data were pre-processed in the Neuroscan software (Neuroscan 4.5, Neuroscan, El Paso, TX, USA) in the following steps: 1) zero-phase shift notch filtering (49–51 Hz) using a finite impulse-response filter with a slope of 24 dB/octave; 2) zero-phase shift band-pass filtering (1–80 Hz) using a finite impulse-response filter with a slope of 12 dB/octave; 3) blinded visual inspection of data quality for all channels using linked-ear reference. Channels with abnormal signals were discarded and replaced by signals interpolated from neighbouring electrodes; 4) Re-referencing to the average electrode.

### Spectral analysis

EEG amplitudes were calculated using the wavelet transform as this has a better time-frequency resolution than the more common Fourier transform [13]. A software package for Matlab 2012a (The Matworks, Inc., Natick, MA, USA) was used. The continuous wavelet transform was applied to EEG from each channel using the complex Morlet wavelet as a mother wavelet function with a bandwith of 10 Hz and a centre frequency of 1 Hz. Scales for the mother wavelet was chosen to match frequencies ranging from 1 to 32 Hz with a 0.5 Hz between-scale frequency interval. The absolute values of the obtained wavelet coefficients were then used for the following analysis: First, the wavelet coefficients were divided into the following standardized bands: delta (1–4 Hz), theta (4–8 Hz), alpha (8–12 Hz) and beta (12–32 Hz). Next, the wavelet coefficients were averaged over time and scales contained within each frequency band were summed together to yield the absolute activity within each frequency band. Finally, the relative activity was calculated separately for each channel by dividing each frequency band with the total energy of all bands and multiplying by 100. The values then represent the percentage of total amplitude contained in each frequency band.

### Functional connectivity

Spectral analysis reduces the complex data from the EEG to simples measures of rhythmicity and therefore does not assess the complex connections between electrodes [9]. Functional connectivity can assess complex brain networks, and has been used within structural and functional magnetic resonance imaging, but is now finding use within EEG research as well [14]. Functional connectivity considers the brain as a complex network of inter-connected nodes rather than assessing the rhythmicity of each node individually [14]. Several methods exist for estimation of functional connectivity within EEG analysis and most methods work by investigating the phase-relationships between EEG signals for different electrodes, where electrodes with similar phases are thought to be exchanging information [15]. The phase-lag index (PLI) is based on a consistent lag between instantaneous phases between two signals and thus ignores most zero-phase phase-relations in order to discard volume conduction noise, and was therefore selected for the current study [15].

The PLI was calculated for the same frequency bands as used for the spectral analysis, using the implementation from The Neurophysiological Biomarker Toolbox (NBT) (http://www.nbtwiki.net/). The signal was divided into time windows and the PLI was calculated for each window. Lastly, the results for all time windows were averaged. The window width was set to twice the sampling frequency (2000 samples) as this provides a frequency resolution of 0.5 Hz, which is equal to the spectral analysis. The band-pass filter was a 1^st^ order butterworth filter [16].

### Statistical analysis

Data are presented as mean±SD unless otherwise indicated. Single channel EEG parameters (amplitudes and PLI) were analysed using a repeated measures ANOVA with the experimental condition (painful vs. control vs. resting) and EEG channels as within-subject factors. The analysis was corrected using the Greenhouse-Geisser method. A post-hoc tukey HSD was performed in the experimental condition or and interaction between experimental condition and EEG channel was present. Correlations were investigated for channels where differences were present between the painful and control condition, using a Pearson’s linear correlation coefficient. The average were taken for all significant channels in a frequency band, and correlated to the reported pain. A P-value below 0.05 was considered statistically significant.

## Results

Out of 175 patients screened for the study 63 were excluded (24 patients declined to participate, 1 was below 18 years of age, 1 was already participating in another study, 1 due to replacement of both hips in the same surgery, 1 because informed consent could not be obtained, 13 for planned spinal anaesthesia during surgery, 4 due to high preoperative opioid use, 1 due to drug abuse and 17 due to a history of neurological conditions). Out of the 112 patients who were eligible for the study and signed the informed consent, 85 were included in the final analysis (15 excluded because surgery was cancelled or consent was withdrawn and 12 patients for having one or more unusable EEG recordings due to poor quality). Clinical and demographic characteristics for the 85 patients are reported in Table 1.

**Table 1:** Demographic and clinical characteristics of patients scheduled for total hip replacement. Since some patients had missing data, the percentage of available is given in a separate column for each parameter. Measures for chronic pain severity are included as the chronic pain grade and MPSS, while severity of arthritis is included as the WOMAC.

		Available data, n (% of total cohort)
Age (years)	64.9 ± 12.1	88 (100%)
Male sex, n (%)	41 (47%)	88 (100%)
BMI (kg/m^2^)	28.1 ± 5.1	85 (97%)
WOMAC	53.8 ± 18.6	70 (80%)
Hip-pain duration, n (%)		88 (100%)
- 0–6 months	14 (16%)	
- 6–12 months	12 (14%)	
- 1–2 years	19 (22%)	
- 2–5 years	24 (27%)	
- More than 5 years	19 (22%)	
Chronic pain grade, n (%)		85 (97%)
- Grade I-II	36 (42%)	
- Grade III-IV	49 (58%)	
MPSS, n (%)		88 (100%)
- Stage I	38 (43%)	
- Stage II	29 (33%)	
- Stage III	21 (24%)	
Preoperative MQS—non-opioids	5.3 ± 4.5	87 (99%)
Preoperative MQS—opioids	0.6 ± 1.6	88 (100%)

WOMAC = Western Ontario and McMaster Universities Osteoarthritis Index; MPSS = Mainz Pain Staging System; MQS = Medication Quantification Scale.

### Hip rotation test

Forty-eight patients (55%) had pain elicited by internal hip rotation; two patients by external hip rotation (2%), while 38 patients (42%) had pain elicited by flexion of the hip. The average pain intensity during movement of the affected hip was 5.5 ± 2.0 NRS, which was significantly more compared to pain scores during movement of the unaffected hip 0.5 ± 1.2 NRS (*P* < 0.001). In fact, 74 patients (84%) reported no pain during rotation of the unaffected hip.

### Spectral analysis

**Fig 1:**
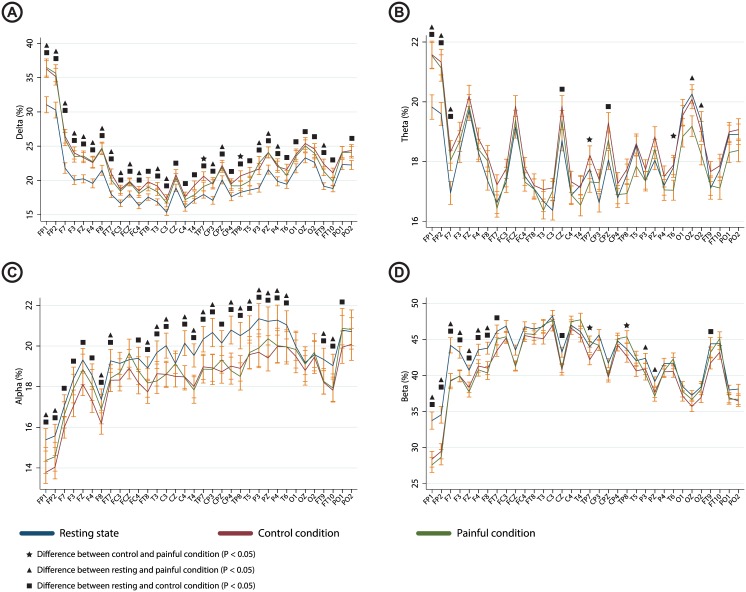
Graph showing the spectral indices under the resting state, rotation of the unaffected hip and rotation of the affected hip for each electrode with indication of significant differences based on the post-hoc test. Values are shown for A) delta band; B) theta band; C) alpha band and D) beta band. Error bars indicate standard error.

**Fig 2:**
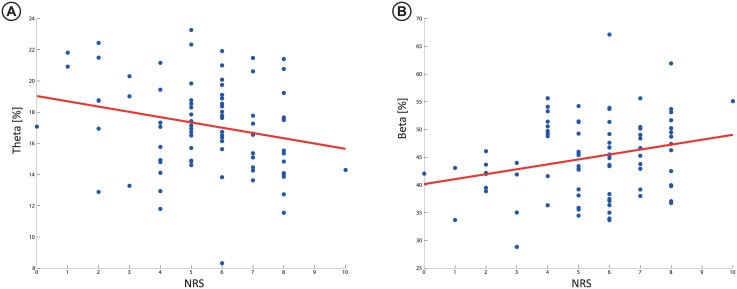
Correlations between the NRS and A) Theta band and B) Beta band indices. The correlation line is shown, indicating the relationship of the correlation.

*Statistical comparison for all electrodes*: Differences within recording conditions were seen for the delta (P < 0.001), theta (P = 0.03), alpha (P = 0.001) and beta (P < 0.001) bands. Values for individual electrodes and the results from the post-hoc test are shown in Fig 1. Post-hoc test revealed differences between painful and control condition in delta, theta and beta. Reported pain during hip rotation were correlated to the channels revealed by post-hoc analysis in the theta (r = -0.24, P = 0.03) and beta (r = 0.25, P = 0.02) bands. The raw correlations are presented in Fig 2

**Fig 3:**
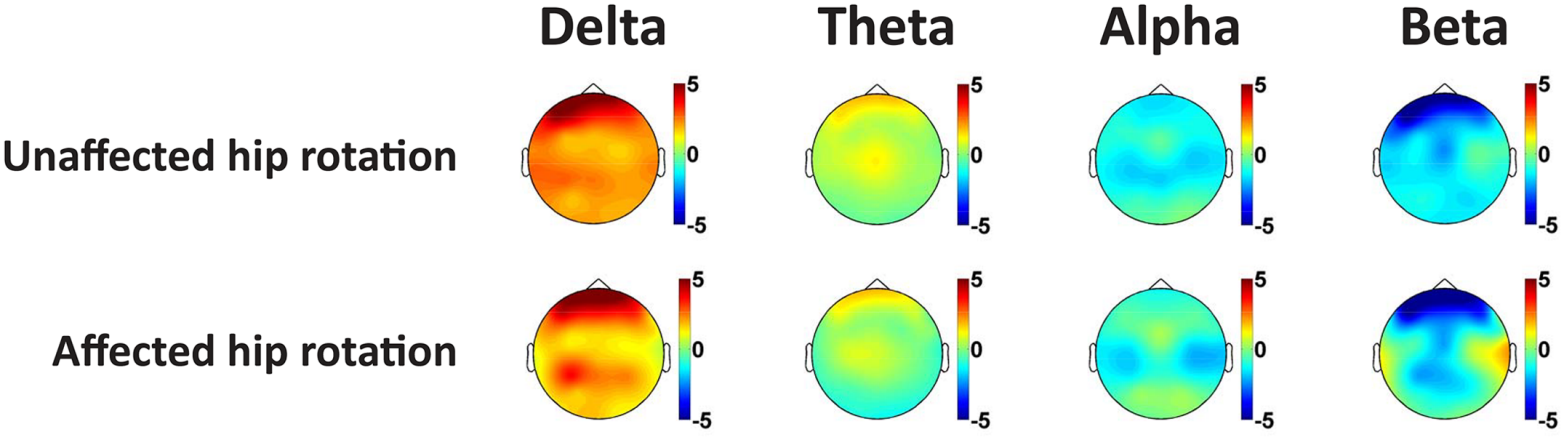
Topographical representation of the differences in relative EEG spectral content during rotation of the unaffected hip and rotation of the affected hip when subtracted the resting EEG as baseline. Warm colours indicate increases in spectral content compared to resting EEG, while cold colours indicate decreases.

Compared to the *resting state* condition, a slowing of the EEG was seen during the painful and control hip rotation conditions. This was evidenced by increased theta and delta EEG amplitudes, with corresponding decreases in the alpha and beta amplitudes. *Topographical comparisons*: The topographical illustration of the differences in spectral content between resting EEG, EEG recorded during rotation of the affected and the control condition is shown in Fig 3. Visual inspection of the topographical plots show a frontal increase in the delta band as well as a decrease in the beta band during both the painful rotation and control condition.

### Functional connectivity

**Fig 4:**
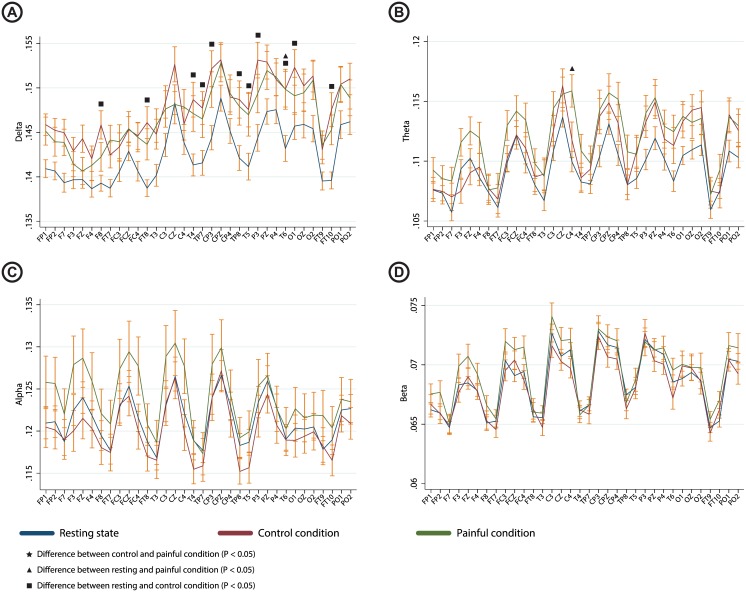
Graph showing PLI under the resting state, rotation of the unaffected hip and rotation of the affected hip for each electrode with indication of significant differences based on the post-hoc test. Values are shown for A) delta band; B) theta band; C) alpha band and D) beta band. Error bars indicate standard error.

Recording condition was a significant factor in the delta (P = 0.003) and theta (P = 0.05) bands. Values for individual electrodes and results from the post-hoc test are shown in Fig 4.

**Fig 5:**
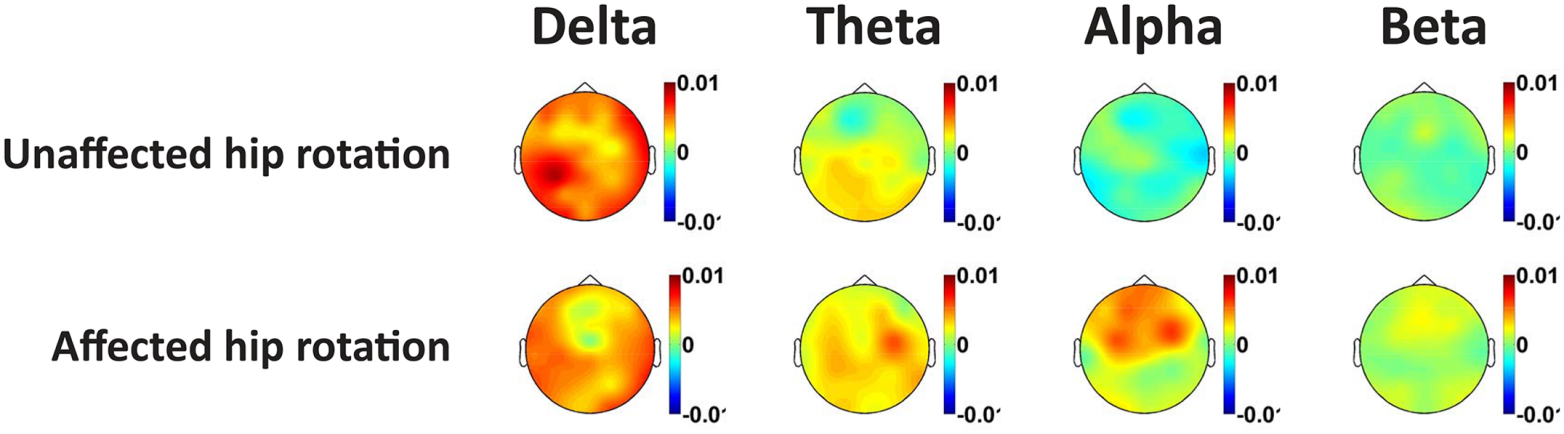
Topographical representation of the differences in phase-lag index during rotation of the unaffected hip and rotation of the affected hip when subtracted the resting EEG as baseline. Warm colours indicate increases in connectivity compared to resting EEG, while cold colours indicate decreases.

Mainly, increased PLI were seen during the control condition compared to resting state in the delta band. The topographical representation is shown in Fig 5.

## Discussion

This study investigated the cortical response, as assessed by multidimensional EEG analysis, to evoked *clinical pain* by means of controlled hip rotations in people with osteoarthritis scheduled for total hip replacement surgery. Differences were seen in the spectral EEG following rotation of both the affected hip (painful condition) as well as the non-affected hip (control condition). Differences were also seen (although to a lesser degree) in the cortical response between the two hip rotation conditions (i.e. painful vs. non-painful), and these differences were correlated (although weakly) to the pain reported during hip rotation. These findings, indicates at connection between the degree of reported pain during evoked clinical pain, and the spectral EEG response. Differences were also seen in the connectivity of the EEG, indicating some change in network characteristics. However, differences were almost exclusively seen between resting and the control condition. This indicates that the changes in network connectivity as related to other factors than the feeling of pain.

### Methodological considerations

The spectral analysis in this study used the continuous wavelet transform. This method has a better temporal resolution and is less sensitive to noise compared to the more commonly used Fourier transform [13]. A novel measure for assessment of functional connectivity, the phase-lag index, was used as it has been shown to be largely resistant to volume conduction [14], which can interfere with signals as opposed to more simple measures such as coherence [17].

The sequence of EEG recordings in this study was fixed; first EEG during resting state was recorded, next EEG during painful hip rotation was performed and lastly EEG recording during hip rotation of the non-affected hip was performed. It was necessary to use a fixed protocol to establish the exact rotation paradigm necessary to evoke pain in the affected hip prior to applying it on the unaffected side. The fixed sequence paradigm may introduce long-lasting effects caused by the painful stimulation in the EEG recorded during the control condition. However, this effect is not specific to the sensation of pain, since there was almost no sensation of pain during rotation of the unaffected hip.

The stimulus in this study was evoked by rotating the hip of patients scheduled to undergo total hip replacement surgery. The pain stimulus in this case is less precise compared to conventional experimental pain stimuli (e.g. heat, pressure and electric stimulation) since it is dependent on the clinical condition of the individual patient, which may introduce a high degree of inter-individual variability in the controlled stimulus. However, the stimulus and nociceptors involved are clinically relevant and may therefore provide information which would not be possible with conventional tonic pain models such a cold/heat pain.

Many studies have used experimental pain models to objectively investigate changes in pain processing underlying acute and chronic pain disorders. Thereby confounding factors connected to the clinical condition, including social, psychological and cognitive aspects of the disease can be minimized [18]. The current study suffers from these confounding factors due to lack of healthy subject since the patients have been suffering from chronic pain with an accompanying decrease in quality of life [19]. When comparing the results of the current study to results from a previous study on healthy volunteers using a tonic painful stimulus (the cold pressor test) with the same methods for analysis [20], the painful stimulus in the current study evoked less changes in EEG activity. However, even though the difference in the current study is smaller, the general trend is the same with increase in delta activity and decreased alpha. This could be due to the confounding factors found in patient populations, and possibly also due to increased brain activity due to motor and proprioceptive brain activation in the current pain model while rotating the hip.

Many recent studies have pointed to the gamma band as a frequency band of interest when dealing with EEG and pain perception [21,22]. This study did not include the gamma band due to high-frequency EMG artifacts induced during the painful tonic stimulation. Including the gamma band while riddled with noise would have affected all of the frequency bands due to the relative content being of interest in this study. Even so, the lack of gamma band data is to be considered a limitation of this study.

### Brain response to pain

Brain responses to painful stimuli have been investigated extensively using various methods such as functional magnetic resonance imaging and EEG based methods including evoked brain potentials. Many studies have revealed an array of cortical and subcortical areas involved in pain processing subsequently named the *pain matrix* [23]. Due to the strong correlation between *pain matrix* activity and the intensity of the stimulus used to evoke experimental pain as well as the perceived pain (subjective pain reporting), it was hypothesized that activation of the pain matrix was distinct and essential for pain perception [23]. However, recent studies have shown that pain perception and *pain matrix* activation can be disassociated under certain experimental paradigms, thus questioning the connection between activation of the *pain matrix* and pain perception [6]. It is speculated that a large part of brain activity in the *pain matrix* can actually be attributed to the saliency of the stimulus [6,23]. This development is similar to the findings in the current study, in that large amount of differences found in the EEG between the resting condition and clinical pain condition are non-specific to pain. However, since the clinical stimulation is tonic, it is likely not attributed to saliency alone. Further experiments are needed to properly determine the cause of these findings.

With advanced methods emerging for EEG analyses, investigations using continuous EEG, e.g. during resting or tonic pain, have become increasingly common. Most studies on tonic pain have reported decreased alpha activity and increased beta activity during tonic cold pain [20,24–26]. This is partially in concurrence with the current study, where alpha activity also decreased during tonic pain. However, beta activity decreased which is in contrast to previous findings. It is not possible to expose the exact reason for this discrepancy, but it could be speculated that the relatively high age of the population in this study could explain the difference since increased age has been shown to induce widespread slowing of the EEG [27,28].

The current study found differences in EEG activity between resting state and painful rotation of the hip in patients scheduled for hip replacement surgery. Differences were also found between the painful condition and the non-painful condition and although the differences were numerically small. If a control condition had not been included in the study, it could be tempting to conclude that the observed difference in the EEG was mainly caused by pain elicited by hip rotation. This clearly underlines the need for a control condition in pain studies investigating pain EEG, as results can easily be interpreted incorrectly without. In experimental thermal tonic pain models, a control condition with an innocuous temperature is not always part of the experimental procedure [20,25]. However, more studies are including the control condition, and immersion of the hand in water with innocuous temperature has been shown to induce differences in the EEG compared to resting state [10,26,29]. With clinical pain the use of control conditions are rare, in many cases because inclusion of a valid control condition can be difficult especially considering visceral or deep pain. In line with this argument our control condition had relevant limitations. In a subgroup of patients rotation on the less or unaffected side was painful, which has to be suspected in hip osteoarthritis that quite commonly affects both sides. Thus the small differences between the two conditions may be caused by bilateral disease, even if there was a relevant difference in clinical pain.

### Conclusions

This study shows that the EEG effects evoked by painful rotation of the hip in patients scheduled for hip replacement surgery could contain pain related activation. This is supported by the correlation between EEG activity and reported pain. Furthermore, the large differences when rotation the other hip which evoked no or minor pain, underlines the importance of including proper control conditions in EEG studies with the aim to investigate pain mechanisms in evoked clinical pain, to reduce the risk of misinterpretation of results.
